# Removal of Mucus from Cervical Canal

**Published:** 1874-12

**Authors:** 


A Syringe for the Removal of the Mucous Plug in the Cervix. Dr. Thomas exhibited a syringe with a long nozzle and a piece of rubber tubing projecting half an inch attached to the nozzle, for the purpose of withdrawing the tenacious mucous plug, frequently obstructing the cervical canal, which often cannot be removed by an ordinary syringe. The end of the tubing is introduced as far as the internal os, and it is surprising into how small a cervical canal it can be passed.—*Proceedings Obstetrical Society of New York.*

